# Simple clotting test to detect procoagulant abdominal swabs

**DOI:** 10.1007/s10856-015-5430-6

**Published:** 2015-02-11

**Authors:** Stefanie Krajewski, Tanja Nathan, Bernd Neumann, Sebastian Hoffmann, Martin Abel, Annette Koggel, Christian Schlensak, Hans P. Wendel

**Affiliations:** 1 Clinical Research Laboratory, Department of Thoracic, Cardiac and Vascular Surgery, University Hospital Tuebingen, Tuebingen University, Calwerstr. 7/1, 72076 Tūbingen, Germany; 2 Seh Consulting + Services, Paderborn, Germany; 3 Medical & Regulatory Affairs, Lohmann & Rauscher GmbH & Co KG, Rengsdorf, Germany

## Abstract

**Abstract:**

During surgical procedures, abdominal swabs are routinely used to adsorb blood from the operation field and for the retention of tissues and organs. Due to the material characteristics, abdominal swabs exhibit a slight procoagulant activity, which is usually desirable and mostly harmless. However, during cardiac surgery with heart–lung machine (HLM) support, abnormal clot formation may result in life-threatening thromboembolic complications. Therefore, a simple clotting test (SCT) allowing in vitro detection of abdominal swabs with elevated hypercoagulant potency in the presence of heparinized human blood was developed and validated. In order to establish a SCT, heparinized human blood from 100 donors was incubated with five different cotton abdominal swabs for 30 min at 37 °C and then macroscopically analyzed. In a second study, 10 other swabs were screened with the established SCT (n = 11) to confirm its suitability. Scanning electron microscopy, measurements of activated clotting times and thrombin-antithrombin were further performed. In the SCT, the results are dichotomized as negative (no detectable blood clot) and positive (blood clot formation). In the first study, three of the five tested abdominal swabs exhibited hypercoagulant potency in at least 25 % of the donors. Calculations using the binomial distribution showed that blood of 11 donors is needed for routine testing with the SCT, which was confirmed in the second study using another 10 swabs. The established SCT can be used for detection of abdominal swabs with an elevated procoagulant potency, thereby minimizing the risk of thromboembolic complications during cardiac surgery with HLM support.

**Graphical Abstract:**

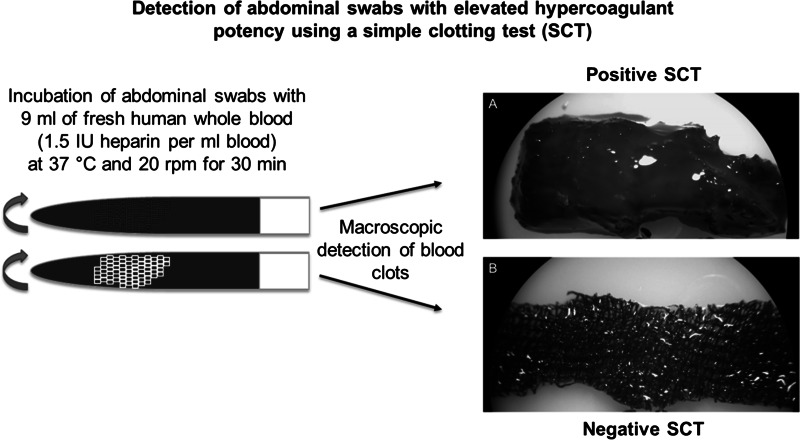

## Introduction

Abdominal swabs are used in surgical routine to adsorb blood and other body fluids from the operation field and for tissue retention.

Upon the occurrence of heavy bleeding during surgery, the blood is sucked using a vacuum blood sucker and disposed off or washed by automated autotransfusion and then retransfused to the patient [1, 2].

In aortocoronary bypass surgery with heart–lung machine (HLM) assistance direct blood retransfusion using a vacuum blood sucker without automated autotransfusion is commonly practiced, because blood coagulation is almost completely absent due to the high anticoagulation (~3 IU heparin/ml blood). Direct retransfusion could in theory be risky due to the use of hypercoagulant abdominal swabs, although the filter and defoamer in the cardiotomy reservoirs provide a further precautionary measurement to eliminate clots.

Abnormal clot formation during complex cardiac surgery on the surface of abdominal swabs in the operation field, may, if retransfused to the extracorporeal circuit, result in life-threatening thromboembolic complications [3]. Hence, the use of abdominal swabs exhibiting excessive prothrombotic potency can result in severe complications during cardiac surgery with HLM support.

This study validates a new simple clotting test (SCT) allowing quick and robust testing of abdominal swabs with heparinized human whole blood before clinical application.

## Materials and methods

### Blood sampling

Blood sampling procedures were approved by the ethic committee of the University of Tuebingen, Germany. Human whole blood was collected from healthy volunteers in monovettes preloaded with heparin (Ratiopharm GmbH, Ulm, Germany) resulting in a heparin concentration of 1.5 IU/ml, which is a maximum of 50 % of the heparin concentration typically used during cardiac surgery under HLM support.

Blood donors, who took hemostasis-affecting agents during the last 2 weeks before blood sampling, were excluded.

### Study 1: validation of the SCT

For each test, pieces of 10 × 40 mm were cut from five cotton abdominal swabs (Table 1) and inserted into sterile 14 ml polypropylene tubes (BD Falcon, Franklin Lakes, USA) followed by addition of 9 ml fresh human whole blood (n = 100). An empty tube containing human blood only served as negative control.

**Table 1:** Overview of cotton abdominal swabs used in study 1 (swab 1–5) and study 2 (swab 6–15) in order to establish and validate the SCT

Swab	Test swabs	Lot	Company	Number tested	Positive SCT	Negative SCT
Study 1						
1	Green (with PDAA)	14128682	Lohmann & Rauscher	n = 100	**n** **=** **97**	**n** **=** **3**
2	White (without PDAA)	22128001	Lohmann & Rauscher	n = 100	n = 2	n = 98
3	Green (without PDAA)	22528002	Lohmann & Rauscher	n = 100	**n** **=** **25**	**n** **=** **75**
4	Blue (without PDAA)	30918015	Lohmann & Rauscher	n = 100	n = 4	n = 96
5	Green (with PDAA)	21458000	Lohmann & Rauscher	n = 100	**n** **=** **76**	**n** **=** **24**
Study 2						
6	White	32658003	Lohmann & Rauscher	n = 11	n = 0	n = 11
7	White	22328012	Lohmann & Rauscher	n = 11	n = 0	n = 11
8	White	22128001	Lohmann & Rauscher	n = 11	n = 0	n = 11
9	White	2473W004	Lohmann & Rauscher	n = 11	n = 0	n = 11
10	Green	22018008	Lohmann & Rauscher	n = 11	**n** **=** **2**	**n** **=** **9**
11	Green	12265182	Mölnlycke Health Care	n = 11	n = 0	n = 11
12	White	102038390	Hartmann	n = 11	n = 0	n = 11
13	Green	299201003	Hartmann	n = 11	n = 0	n = 11
14	Green	30668	NOBA	n = 11	n = 0	n = 11
15	Green	3000316	Lohmann & Rauscher	n = 11	n = 0	n = 11

Abdominal swabs with procoagulant activity are highlighted in bold

*PDAA* polydiallyamine; stabilizer for colors

To simulate slight blood flow and body temperature as observed in the abdominal operation field, the samples were incubated for 30 min at 37 °C on an orbital shaker (Polymax 1040, Heidolph, Schwabach, Germany) at 20 rpm. Directly after incubation, all samples were carefully rinsed with saline and the swabs were macroscopically analyzed with regard to blood clot formation.

### Study 2: screening of different abdominal swabs using the SCT

A total of another 10 abdominal swabs were analyzed in the SCT as described above.

Furthermore, activation of blood coagulation was measured before and after contact of two abdominal swabs with fresh human whole blood, which was anticoagulated with various heparin concentrations ranging from 0.75 to 1.5 IU/ml. As a plasmatic marker for coagulation, we determined thrombin–antithrombin-III complexes (TAT) with an enzyme-linked immunosorbent assay (Siemens Healthcare, Marburg, Germany).

### Activated clotting time (ACT)

ACT measurements were performed directly after blood taking using the Hemochrom Jr. II system (Life Systems, Hamburg, Germany) [4].

### Scanning electron microscopy (SEM)

SEM was performed to investigate the thrombogenicity of the swabs after contact with whole blood as previously described [5].

### Statistics

The SCT was validated in study 1 with a sufficiently large and randomly selected panel of healthy donors. As results are dichotomized as negative (no blood clot formation) and positive (blood clot formation), the sample size calculation focused to obtain estimates of the probability of detecting a swab expected to be positive/negative with sufficient precision, i.e. with a maximum confidence interval length of 5 % for positive swabs and 10 % for negative swabs. Based on the expectation that at least 97.5 % of donors would react to a positive swab and at least 95 % of donors would not react to a negative swab, the mid-p method according to Fosgate [6] resulted for the significance level of 5 % in 94 and 46 donors, respectively. Choosing the larger of these two and adding a margin to compensate for potential dropouts, a sample size of 100 donors was used.

The number of donors, i.e. n = 11, to be used in study 2 and potentially for routine testing was determined by calculations using the binomial distribution. Confirmation of the suitability of the SCT was performed by testing 10 different abdominal swabs with the blood of 11 healthy donors.

## Results

### Study 1: development and validation of a reliable and fast test method to detect abdominal swabs with hypercoagulant potency

In order to establish a reliable, easy and rapid test for the detection of coagulation properties of abdominal swabs, which are routinely used during surgery, a dynamic in vitro model using heparinized human blood of a large, randomly selected panel of 100 donors was used.

The blood was anticoagulated with a final heparin concentration of 1.5 IU/ml resulting in a mean ACT value of 313.6 ± 43.4 s.

Five test cotton abdominal swabs (Table 1, swabs 1–5) were used in order to demonstrate the robustness and usefulness of the method, whereby abdominal swab number 1 was expected to be positive (bad colored and with stabilizer for colors, polydiallyamine (PDAA)), while the white swab without color was expected to be negative in the SCT. The other three colored test swabs (with and without PDAA) may theoretically exhibit different procoagulant potencies.

After incubation for 30 min, the 5 abdominal swabs tested were macroscopically examined with regard to blood clot formation.

**Fig. 1:**
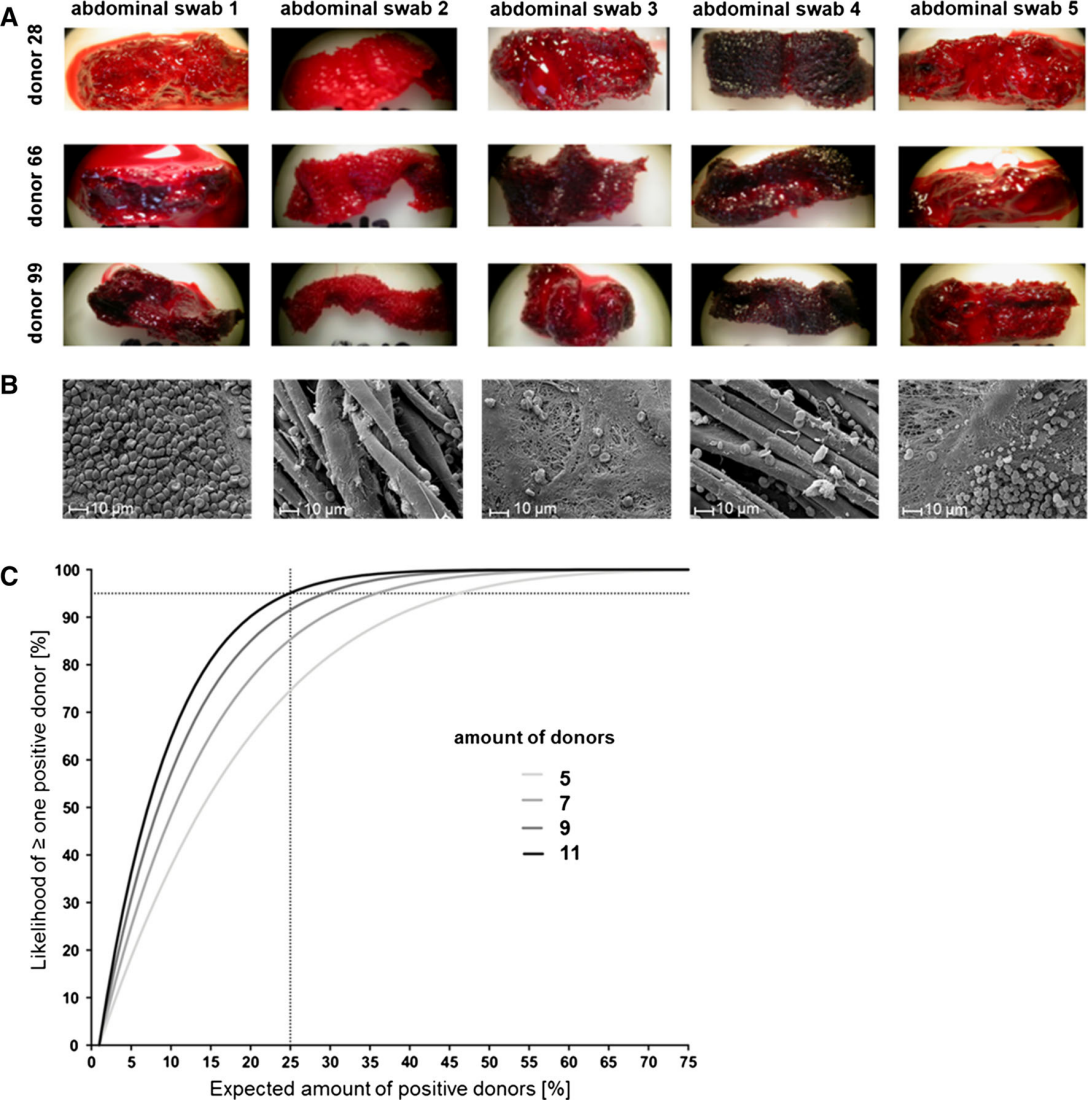
**a** Representative macroscopic images of five different cotton abdominal swabs tested in study 1 after incubation with heparinized human whole blood of 3 donors in the SCT. **b** Representative SEM images of the five abdominal swabs after incubation with whole blood for 30 min at 37 °C; magnification ×2,500. **c** Likelihood of detecting a positive swab in dependence of expected proportion of positive donors (0–100 %) and amount of donors (*5*; *7*; *9*; *11*). The *dotted lines* indicated that in order to detect a swab to which 25 % of donors react with 95 % likelihood 11 donors need to be tested

Representative macroscopic images of 3 donors are shown in Fig. 1a for all 5 abdominal swabs. In total, blood clotting upon contact of the abdominal swabs with blood of 100 different healthy donors was analyzed each under blinded conditions by three examiners. The results show that the abdominal swabs number 2 and 4 only induced blood clotting in 2 and 4 %, respectively, whereas no blood clotting was observed in the other 98 or 96 % of healthy donors tested (Table 1, swabs 1–5). As expected, the abdominal swab number 1 exhibited a strong hypercoagulant activity with blood clotting in 97 % of the donors tested.

Interestingly, a lower, but still existent, procoagulant potency was observed after incubation of human whole blood with the abdominal swab number 5, where blood clotting was observed in 76 % of the donors. Also, abdominal swab number 3 induced blood clotting in 25 % of the donors. Furthermore, SEM images of each abdominal swab after contact with heparinized human whole blood are exemplarily shown from one donor at magnifications of ×2,500 (Fig. 1b). Again, the abdominal swabs number 1, 3 and 5 showed an increased procoagulant potency indicated by the profound adhesion of blood cells, particularly platelets and red blood cells, and the formation of a dense fibrin network. In contrast, only a few blood cells were detected on the surface of the abdominal swabs number 2 and 4 indicating good hemocompatible surface properties.

Statistical analysis of the macroscopic results was carried out independently in order to deduce the amount of donors needed for routine testing. As demonstrated in Fig. 1c, which shows the likelihood of at least one positive donor in dependence of the amount of donors and expected proportion of reacting donors, 11 donors are required to detect a swab that induced positive reactions in 25 % of donors.

### Study 2: screening of different abdominal swabs using the novel SCT

In order to verify the SCT, another 10 cotton abdominal swabs (Table 1, swabs 6–15) were analyzed using heparin anticoagulated blood of 11 healthy donors.

The results indicate that only one of the 10 tested swabs, i.e. abdominal swab number 10, exhibits hypercoagulant activity in 2 cases. All other swabs did not induce blood clotting and can therefore be safely used during cardiac operations (Table 1, swabs 6–15).

In order to verify the results gained from macroscopic analysis of blood clotting in the SCT, TAT plasma concentrations were exemplarily analyzed after incubating abdominal swabs number 1 and 7 with blood anticoagulated with 0.75, 1, 1.25 or 1.5 IU/ml heparin (data not shown). At a heparin concentration of 1.5 IU/ml, a TAT concentration of 4.89 ± 0.6 µg/l was measured in the samples incubated with the white abdominal swab (number 7), whereas the green abdominal swab number 1 induced a significantly higher TAT complex formation resulting in a value of 1,449.17 ± 499.2 µg/l after 30 min of incubation.

In summary, our results show that 4 of the 15 tested abdominal swabs in study 1 and 2 exhibit procoagulant potency and should hence not be used during cardiac operations with HLM support.

## Conclusion

It is deemed necessary to establish a quality control system to detect abdominal swabs having abnormal coagulatory activity in the presence of heparinized blood to eliminate the risk of thrombotic complications during cardiac surgeries under HLM support. We suggest to implement the SCT in industrial manufacturing processes as a routine quality assurance procedure to identify a potential hypercoagulant activity of an abdominal swab in 25 % of the 11 blood donors with a probability of 95 %. At the current level of knowledge, the use of abdominal swabs with hypercoagulant activity during surgical applications other than in cardiac surgery with HLM support poses no risk.
